# Airway regulatory T cells are decreased in COPD with a rapid decline in lung function

**DOI:** 10.1186/s12931-020-01593-9

**Published:** 2020-12-14

**Authors:** Jonas Eriksson Ström, Jamshid Pourazar, Robert Linder, Anders Blomberg, Anne Lindberg, Anders Bucht, Annelie F. Behndig

**Affiliations:** 1 Department of Public Health and Clinical Medicine, Division of Medicine, Umeå University, 90187 Umeå, Sweden; 2 Division of CBRN Defence and Security, Swedish Defence Research Agency, Stockholm, Sweden

**Keywords:** Chronic obstructive pulmonary disease, Disease mechanisms, Lung function decline, Smoking habits, Bronchoalveolar lavage, Regulatory T cells

## Abstract

**Background:**

Differences in the expression of regulatory T cells (Tregs) have been suggested to explain why some smokers develop COPD and some do not. Upregulation of Tregs in response to smoking would restrain airway inflammation and thus the development of COPD; while the absense of such upregulation would over time lead to chronic inflammation and COPD. We hypothesized that—among COPD patients—the same mechanism would affect rate of decline in lung function; specifically, that a decreased expression of Tregs would be associated with a more rapid decline in FEV_1_.

**Methods:**

Bronchoscopy with BAL was performed in 52 subjects recruited from the longitudinal OLIN COPD study; 12 with COPD and a rapid decline in lung function (loss of FEV_1_ ≥ 60 ml/year), 10 with COPD and a non-rapid decline in lung function (loss of FEV_1_ ≤ 30 ml/year), 15 current and ex-smokers and 15 non-smokers with normal lung function. BAL lymphocyte subsets were determined using flow cytometry.

**Results:**

The proportions of Tregs with regulatory function (FoxP3^+^/CD4^+^CD25^bright^) were significantly lower in COPD subjects with a rapid decline in lung function compared to those with a non-rapid decline (p = 0.019). This result was confirmed in a mixed model regression analysis in which adjustments for inhaled corticosteroid usage, smoking, sex and age were evaluated. No significant difference was found between COPD subjects and smokers or non-smokers with normal lung function.

**Conclusions:**

COPD subjects with a rapid decline in lung function had lower proportions of T cells with regulatory function in BAL fluid, suggesting that an inability to suppress the inflammatory response following smoking might lead to a more rapid decline in FEV_1_.

*Trial registration* Clinicaltrials.gov identifier NCT02729220

## Background

Despite many efforts to understand the pathophysiology of COPD, it is still unknown why some long-term smokers develop COPD and some do not. One proposed explanation is differences in the ability to regulate the immunological response to inhalation of tobacco smoke [1]. Upregulation of regulatory immune cells would restrain airway inflammation and, thus, the development of COPD; while the absence of such upregulation would over time lead to chronic inflammation and eventually COPD.

In COPD, airway inflammation is characterized by increased numbers of neutrophils and macrophages [2]. Macrophages are thought to play a major role as they can release matrix metalloproteinases capable of degrading the extracellular matrix in the lungs, as well as secrete chemokines that attract other immune cells such as monocytes and lymphocytes. In the latter population, the balance in COPD airways is tipped towards cytotoxic cell types such as CD8^+^ T cells [3] and NK cells [4]. Among regulatory immune cells, regulatory T cells (Tregs) are thought to be a key player, particularly in protecting the body from an over activated immune response [5].

Because of the chronic inflammation observed in COPD, it has been hypothesized that Tregs would be reduced in subjects affected by the disease [5]. In 2008, Barceló et al. showed that smokers with preserved lung function (LF) had a prominent upregulation of Tregs in bronchoalveolar lavage (BAL) compared to COPD subjects [1]. In other studies, however, Tregs have been found to be associated with pack-years rather than COPD status [6] and even to be increased in COPD [7].

In flow cytometry, the most commonly used marker for Tregs has been the intracellular FoxP3 (Forkhead Box P3) transcription factor, but early studies often defined Tregs as CD4^+^ CD25^bright^ cells and some later studies instead opted for CD127^low^ as the defining characteristic. While intended to identify the same cell population, these definitions do not overlap completely and might even reflect different immunological functions. For example, Roos-Engstrand et al. showed that while CD4^+^ CD25^bright^ cells were increased in smokers and COPD, the expression of FoxP3 was not, indicating an expansion of helper T cells without regulatory function in the studied population [8].

The current study is part of the KOLIN (‘Respiratory and Cardiovascular Effects in COPD’) project which was designed primarily to investigate the rapid decline phenotype of COPD. Focusing on regulatory immune cells, the aim was to assess the distribution of Tregs in COPD in general and its association with disease status, smoking status and rapid/non-rapid decline in LF in particular. Findings related to cytotoxic immune cells have been reported previously [4].

## Methods

### Study subjects

52 subjects participated in this cross-sectional study; 12 COPD subjects with a rapid decline in LF (COPD rapid), 10 COPD subjects with a non-rapid decline in LF (COPD non-rapid), 15 current and ex-smokers with normal LF (ever-smokers) and 15 non-smokers with normal LF (non-smokers).

All subjects were recruited from the OLIN COPD study [9] which also provided the longitudinal data used to calculate rate of decline in LF. Rapid decline was defined as a loss of forced expiratory volume in one second (FEV_1_) ≥ 60 ml/year and non-rapid decline as a loss ≤ 30 ml/year, both measured over at least 5 years. The rate of decline was calculated as (FEV_1_ at recruitment − FEV_1_ at follow-up)/number of years of follow-up [10].

To define COPD, the Global Initiative for Obstructive Lung Disease (GOLD) spirometric criteria were used [11]. All COPD subjects and Ever-smokers had a smoking history of at least 10 pack-years. Ex-smokers had stopped smoking at least 12 months prior to inclusion in the study. The recruitment process has been described in detail previously [10].

All subjects underwent bronchoscopy with BAL. Subjects with medical conditions contradicting bronchoscopy and/or inflammatory conditions or medication expected to affect the outcome of the study were excluded from participation. None of the subjects reported exacerbations in the 4 weeks prior to bronchoscopy.

Subject demographics and basic characteristics for COPD subjects are given in Table 1.

**Table 1:** Basic characteristics of COPD subjects, by rate of decline in lung function

	COPD rapid decline in LF n = 12	COPD non-rapid decline in LF n = 10
Female:Male	2:10	4:6
Age^b^	63 ± 7	67 ± 6
BMI^a^	26 ± 3	25 ± 3
Current:Ex-smokers^a^	8:4	3:7
Pack-years^a^	37.5 ± 16	33 ± 11
FEV_1_, percent of predicted^b^	60 ± 15	63 ± 19
FEV_1_/VC^b^	0.52 ± 0.12	0.54 ± 0.11
BAL-recovery, %^c^	44 ± 16	40 ± 19
Annual decline in FEV_1_, ml^b^	86 ± 29	16 ± 17
Use of inhaled corticosteroids; Yes:No^c^	2:10	5:5

Values given as mean ± SD unless indicated differently. Statistical comparisons between the two groups were made using the Mann–Whitney U-test and a *p*-value < 0.05 was considered significant

*NS* Not significant, *Pack-years* (number of cigarettes smoked per day/20) × number of years smoked, *FEV*_*1*_ forced expiratory volume in 1 s, *VC* vital capacity

^a^At time of identification in the OLIN COPD study

^b^At time of inclusion in the current study

^c^At time of bronchoscopy in the current study

### Study design

**Fig. 1:**
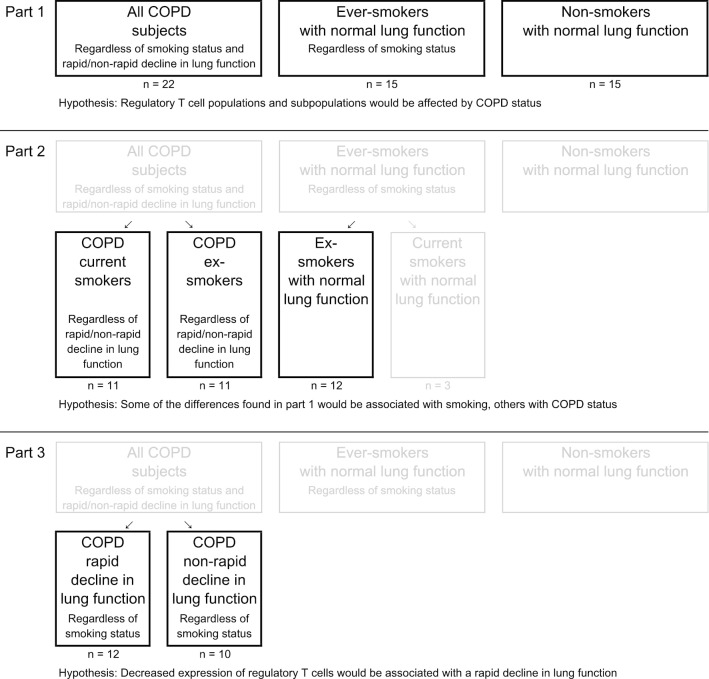
Study design

As all studies part of the KOLIN project, the current study was divided into three parts, each testing a different hypothesis (Fig. 1).

In part 1 the distribution of Treg populations was examined using as big groups as possible. All COPD subjects were compared to ever-smokers and non-smokers. It was hypothesized that COPD would be associated with decreased proportions of Tregs.

In part 2, it was hypothesized that some of the differences found in part 1 would be associated with current smoking and some with COPD. To separate between these, we compared (1) COPD current smokers to COPD ex-smokers; differences between these groups would likely be related to smoking status (since disease status is the same for both groups); and (2) COPD ex-smokers to ex-smokers with normal LF; differences between these groups would likely be related to disease status (since smoking status is the same for both groups).

In part 3, it was hypothesized that a rapid decline in LF would be associated with decreased proportions of Tregs. COPD rapid was compared to COPD non-rapid.

### Spirometry

Following the American Thoracic Society/European Respiratory Society guidelines [12], dynamic spirometry variables were measured using a dry volume spirometer (Mijnhardt Vicatest 5, the Netherlands). If FEV_1_ was lower than 80% of predicted or if FEV_1_/VC was below 0.70, reversibility testing was performed. The highest value out of pre- and post-bronchodilatation FEV_1_ and VC was reported. Swedish spirometric reference values were used [13].

### Bronchoscopy

Bronchoscopies were performed at two locations (the Division of Respiratory Medicine and Allergy, Department of Medicine, Sunderby Central Hospital of Forgotten, Luleå, Sweden and the Division of Respiratory Medicine and Allergy, Department of Medicine, University Hospital, Umeå, Sweden) but by the same medical team. 30 min before the procedure, subjects were given 1.0 mg of atropine subcutaneously. Some also received midazolam 4–8 mg per os. Local anaesthesia was achieved using lidocaine. All subjects were examined in the supine position. BAL was performed by infusing three aliquots of 60 ml of sterile sodium chloride (0.9%), pH 7.3 at 37 °C in the middle lobe or lingula. The fluid was gently sucked back after each infusion and pooled into a tube placed in iced water. The recovered BAL fluid (BALF) was immediately transported to the laboratory for analysis. Bronchial wash (2 × 20 ml) and biopsies were also performed but not included in the analysis in the current study.

In three COPD subjects, BAL could not be performed due to problems tolerating the bronchoscopy procedure. In one COPD subject, BALF recovery was too low to perform flow cytometry analysis.

### Flow cytometry analysis

BALF lymphocyte subsets were determined using a FACSCalibur™ (Becton Dickinson) flow cytometer. BALF cells were centrifuged and adjusted to a final concentration of 10^6^ cells/ml. For each test, different fluorophore-conjugated antibody panels and subtype Ig conjugated with respective fluorophore were combined (Additional file 1: Table S1). Each test tube contained 200–400 μl of cell suspension (10^6^ cells/ml) to which 10 μl of each surface antibody was added and incubated for 30 min at 4 °C in darkness. After the surface marker staining, cells were washed twice with PBS and processed for intracellular staining of FoxP3 using eBioscience fixing and permeabilisation set (Thermo Fisher Scientific, Sweden). Briefly, cells were fixed and permeabilised with staining buffer at 4 °C for 30 min, thereafter washed twice by centrifugation at 4 °C for 10 min, 300*g*. 10 ml FoxP3 antibody was added and allowed to bind for 30 min and thereafter cells were washed twice by adding permeabilisation buffer and centrifuging. Finally, PBS was added for performing analysis using FACSCalibur™ [8]. The lymphocyte population was confirmed by CD45 positive leucocyte population and gated based on the cells’ physical characteristics in a region according to their characteristic forward scatter (FCS) and side scatter (SSC) profiles (Additional file 1: Figure S1). 6000–9000 cells were collected in CD3^+^ gate per test tube and percentage of CD3 subpopulation was counted out of gated lymphocytes and furthermore out of gated subpopulations (Table 2 and Additional file 1: Figure S1). Appropriate isotype-matched controls were used in all experiments. To ensure that autofluorescence did not influence the results, we stained for CD45/CD14 and compared that to the results of lymphocyte and macrophage gating. Flow cytometry data were acquired and analysed using Cell Quest Software (Becton Dickinson). Cell staining and data acquisition were performed at one centralized location.

**Table 2:** Cell populations and FACS staining characteristics

Population	Staining characteristics	When given in percent, calculated as proportion of
T cells	CD3 +	–
T helper cells	CD3^+^ CD4^+^	CD3^+^
Activated T helper cells	CD3^+^ CD4^+^ CD25^bright^	CD3^+^ CD4^+^
FoxP3^+^ regulatory T cells	CD3^+^ CD4^+^ CD25^bright^ FoxP3^+^	CD3^+^ CD4^+^ CD25^bright^

### Statistical analysis

Data was analysed using a two-step approach. In the first step, the investigated cell populations were analysed using group-wise comparisons with no outliers removed. For statistical comparisons between more than two groups, the Kruskal–Wallis test was used and a p-value < 0.05 was considered significant. If the Kruskal–Wallis test indicated significance, the Mann–Whitney U-test was carried out for post-hoc comparison between two groups.

In the second step, cell populations with significant between-group differences were further examined using multivariable mixed effects regression models with no outliers removed. These models were performed by specifying the response variable as the number of cells in the population evaluated (numerator) and the remaining number of lymphocytes (denominator) and incorporating subjects as random effect (random intercepts) in the linear predictor of a generalized linear model with a binomial error distribution. In the mixed effects regression models, adjustments were evaluated for age, sex, use of inhaled corticosteroids and smoking (where applicable). Smoking was evaluated both as a categorical variable (smoking status) and as a continuous variable (pack-years).

Correlation analyses were assessed with Spearman’s ranked test.

Statistical analysis was performed using IBM SPSS Statistics (version 26) and, for calculating mixed effects regression models, statistical software package R (version 3.3.3; R Development Core Team, R foundation for Statistical Computing, Vienna, Austria).

## Results

The study was analysed in three parts (Fig. 1), in part 1 and 2 the aim was the to distinguish the effect of COPD from that of smoking. Group-wise comparisons in these parts revealed no significant differences in Treg populations or in activated T helper cells (Additional file 1: Table S2).

**Fig. 2:**
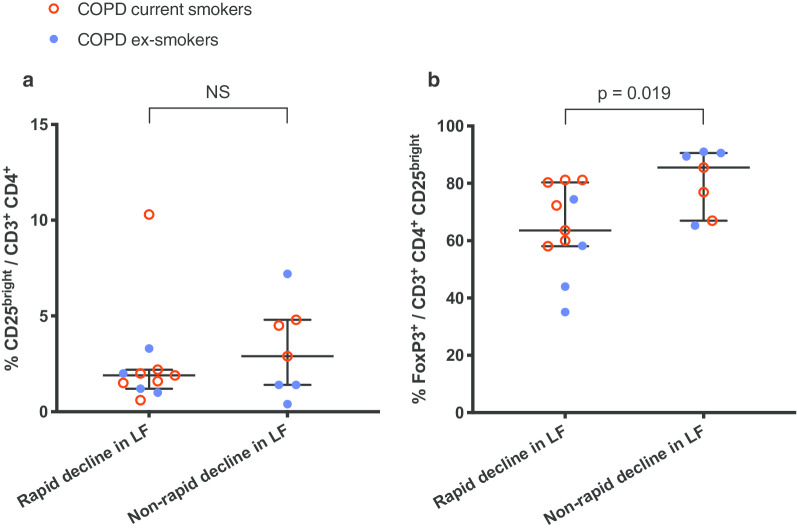
Part 3: Flow cytometry of BAL fluid from COPD subjects. **a** Activated T helper cells in (in percentage of T helper cells). **b** FoxP3^+^ regulatory T cells (in percentage of activated T helper cells). Data shown as median and IQR. *NS* not significant, *LF* lung function. Shown *p*-value calculated using the Mann–Whitney U-test. See Additional file 1 for corresponding data in tables

In part 3, the proportion of FoxP3^+^ regulatory T cells (FoxP3^+^/CD3^+^ CD4^+^ CD25^bright^) was significantly decreased in COPD rapid compared to COPD non-rapid (Fig. 2). The activated T helper cell population (CD25^bright^/CD3^+^ CD4^+^) did not differ significantly between groups (Fig. 2).

The multivariable mixed effects regression model showed a statistically significant relationship between decreased proportions of FoxP3^+^ regulatory T cells and having a rapid decline in LF (COPD rapid vs COPD non-rapid; OR (95% CI) 0.38 (0.19–0.77); p = 0.010). This relationship was significant also after adjusting for current smoking (OR (95% CI) 0.36 (0.18–0.72); p = 0.006) and inhaled corticosteroid (ICS) usage (OR (95% CI) 0.37 (0.18–0.77); p = 0.010).

**Fig. 3:**
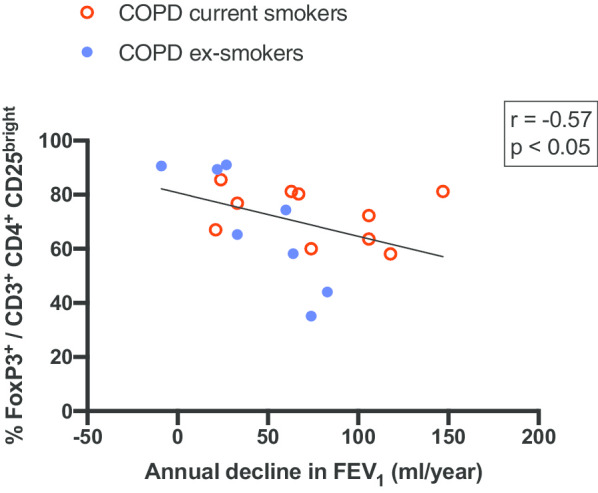
Relationship between FoxP3^+^ regulatory T cells (in percentage of activated T helper cells) and annual decline in FEV_1_ (ml/year). Shown *r*- and *p*-values determined by Spearman’s rank correlation coefficients

A moderate negative correlation was found between the proportion of FoxP3^+^ Tregs in BAL and annual decline in LF (r = − 0.57, p < 0.05; Fig. 3). No correlation was found between pack-years and Treg populations.

## Discussion

### Part 1 and 2—Tregs and COPD

Contrary to our hypothesis in part 1 (Fig. 1), no significant differences in Treg populations were found between COPD subjects and smokers or non-smokers with normal LF. Nor did part 2 reveal any alterations related to smoking or COPD status in these populations.

Previous studies have shown that in COPD, immune cell alterations in one compartment (e.g. peripheral blood) is not necessarily reflected in another (e.g. the lungs) [14]. The distribution of immune cells also differs between lung compartments (small/large airways, lymphoid tissue etc.) [15]. Thus, when evaluating the role of Tregs in COPD, results from studies of one compartment cannot with any certainty be applied to another. As for the bronchoalveolar compartment, there are not many previous studies of Tregs in BAL in relation to COPD.

Barcelo et al. found decreased levels of CD4^+^ CD25^bright^ Tregs in BAL among COPD subjects compared to smokers with normal LF [1]. Smyth et al. found a correlation between pack-years and CD4^+^ CD25^bright^ Tregs in BAL [6]. Neither of these findings were confirmed in the current study.

One possible explanation is dissimilarities in the study populations. Both Smyth et al. and Barcelo et al. recruited their subjects from patients already known to the health care, while our subjects were recruited from the population-based OLIN studies [10]. Since only 20–30% of all individuals with COPD are identified by the health care [16, 17], using these different basis for recruitment would be expected to result in differences in study populations which in turn could affect results.

Another possible explanation could be that the current study is underpowered to detect these differences. As mentioned above, there few previous studies of Tregs and COPD in BAL, making power calculations uncertain.

### Part 3—Tregs and rapid decline

To the best of our knowledge, this is the first study to investigate the role of Tregs in relation to the rapid decline phenotype of COPD. FoxP3^+^ Tregs were found to be significantly decreased in BAL from COPD subjects with a rapid decline in LF compared to COPD subjects with a non-rapid decline. The mixed effects regression analysis showed that this relationship was significant even after adjustments for smoking and use of ICS. Adjusting for the latter is important since ICS usage previously has been shown to lead to upregulation of FoxP3^+^ Tregs [18–20].

Furthermore, a moderate negative correlation was found between FoxP3^+^ Tregs and annual decline in LF. This correlation must however be interpreted with caution given that COPD subjects with an annual decline in LF > 30 and < 60 ml/year were by design not included in the study (as they were defined as not rapid nor non-rapid decliners).

While the hypothesis in part 3 of the study was confirmed and seemingly fits well with the proposed pathophysiological model—that decreased Tregs would lead to unregulated inflammation in response to tobacco smoke and to progression of the disease [1]—this study is cross-sectional and can as such not address the question of causality; if decreased Tregs do indeed lead to a rapid decline in LF or if it is the other way around.

In COPD in general, the decline in LF is thought to be the result of emphysema and airway remodelling. The development of emphysema has traditionally been described as due to infiltration of activated neutrophils leading to an imbalance in protease/antiprotease activity, but more recently additional factors such as oxidative stress and autoimmunity have also been proposed [21]. Remodelling of small airways is also thought to be related to infiltration of neutrophils as well as macrophages and lymphocytes, leading to epithelial thickening and smooth muscle hypertrophy. What these processes have in common is an abnormal inflammatory response to tobacco smoke.

Tregs have the ability to suppress over activated immune responses by contact-dependent mechanisms as well as through the release of cytokines—IL-10 (which has anti-inflammatory effects on neutrophils), IL-35 (which inhibits T-cell proliferation) and TGF-β (which regulate epithelial cells, macrophages and fibroblasts) to name just a few [5].

It therefore seems plausible that an inability to upregulate Tregs could lead to a more rapid development of emphysema and small airway remodelling, and thus to a more rapid decline in LF. However, further longitudinal studies are needed to clarify the role of Tregs in relation to the rapid decline phenotype of COPD.

### Defining rapid decline

As the interest for subtyping is growing within the field of COPD research, it is important to identify which of the proposed phenotypes are clinically relevant. Rapid decline has been described as a separate phenotype of COPD [14, 22] and one with a poor prognosis [23, 24].

There is however no established definition of the term ‘rapid decline’. Many studies have used annual decline in FEV_1_ as the defining characteristic, but the cut-off between rapid and non-rapid decliners in these studies has varied between 40 and 90 ml/year [24–27]. Other studies have opted for using percentiles instead of a fixed cut-off [28], percent of predicted FEV_1_ instead of in FEV_1_ [14] and using Hierarchical Bayesian Models [23] to identify rapid/non-rapid decliners.

To enable an evaluation of whether ‘rapid decline’ is indeed a relevant phenotype of COPD, it would be desirable to reach a consensus on the definition of the term. This should be addressed in further studies of COPD and the role of rate of decline in LF.

### Defining Tregs

As mentioned in the background, there is a plethora of markers available for identifying the Treg population using flow cytometry. A high expression of FoxP3 is considered a hallmark of CD4^+^ Tregs [29], and was the defining characteristic used in the current study. In contrast to some previous studies, the CD3^+^ CD4^+^ CD25^bright^ population was not defined as Tregs as it is likely to encompass activated T cells as well as regulatory ones [30].

Many previous studies of Tregs and COPD rely on blood samples for their analysis. One strength of the current study is that we instead have investigated the main target organ of COPD. Even though COPD is a systemic syndrome [21], when trying to uncover immunological mechanisms affecting the rate of decline in LF, investigating the lungs is likely to generate more relevant data than using peripheral blood.

Another strength is having recruited our subjects using longitudinal data from a well-characterized cohort and the use of a multivariable regression model. The former supplied reliable demographic and other data (i.e. on ICS usage, smoking habits) which then were used in the latter to make necessary adjustments. This enabled us to validate that significant between-group results were indeed associated with the major difference in characteristic and not with other confounding factors.

None of the subjects reported exacerbations in the four weeks leading up to the bronchoscopy. While exacerbations are important to consider in all studies of COPD, they might be of special importance when investigating Tregs as previous studies have shown that this cell population increases in patients with acute exacerbations [31].

One limitation of the current study is that women were underrepresented in the COPD rapid decline and non-smokers groups (Table 1). Thus, we could not evaluate sex-specific differences, nor rule out that results were affected by these differences in group composition. There are previous studies indicating that the links between cellular events and phenotypes of COPD might be different in females compared to males [32]. Oestrogen has been proposed to have a protective role in the development of COPD [33], possibly through stimulating the expansion of Tregs [34].

Data on lymphocyte populations and subpopulations are presented in relative and not absolute cell numbers in the current study. The reason for this is that BAL recovery volumes, as in previous studies [35, 36], were found to be lower in COPD subjects compared to both non-smokers and ex-smokers with normal LF and also to vary within the COPD group [10]. We therefore believe that relative cell numbers better reflect differences in the inflammatory response.

## Conclusions

No significant differences in airway regulatory T-cells were found between COPD subjects and smokers/non-smokers with normal lung function. However, FoxP3^+^ regulatory T cells were decreased in BAL from COPD subjects with a rapid decline in lung function compared to COPD subjects with a non-rapid decline. This result was significant before as well as after adjustments for ICS usage and smoking.

Based on previous research, it seems more plausible that a low expression of regulatory T cells would lead to a rapid decline in lung function, than the other way around. However, as the question of causality cannot be answered in a cross-sectional study such as the current, further longitudinal research is needed to clarify the relationship between regulatory T cells and the rapid decline phenotype of COPD.

## Supplementary information

**Additional file 1: Table S1.** Antibodies conjugated fluorophores, clone and supplier. **Figure S1.** FACS gating strategy. **Table S2.** Flow cytometry analysis of activated and regulatory T cells in BAL fluid, given in percent. **Table S3.** Flow cytometry analysis of activated and regulatory T cells in BAL fluid, given in cells/ml × 10^2^. **Table S4.** Differential cell counts of leukocytes of in BAL fluid, given in number of cells/ml × 10^4^.

## Data Availability

The datasets used and/or analyzed during the current study are available from the corresponding author on reasonable request.

## Abbreviations

BAL: Bronchoalveolar lavage
BALF: Bronchoalveolar lavage fluid
CI: Confidence interval
COPD non-rapid: COPD subjects with a non-rapid decline in lung function
COPDrapid: COPD subjects with a rapid decline in lung function
Ever-smokers: Current and ex-smokers with normal lung function
FACS: Fluorescence-activated cell sorting
FEV_1_: Forced expiratory volume in one second
FoxP3: Forkhead Box P3
GOLD: Global Initiative for Obstructive Lung Disease
KOLIN: ‘Respiratory and Cardiovascular Effects in COPD’
LF: Lung function
Non-smokers: Non-smokers with normal lung function
OLIN COPD study: Obstructive Lung Disease in Northern Sweden Chronic Obstructive Pulmonary Disease study
OR: Odds ratio
Tregs: Regulatory T cells
VC: Vital capacity
