# Supplementary material for: Airway regulatory T cells are decreased in COPD with a rapid decline in lung function
Source: Respir Res. 2020 Dec 14;21:330. doi: 10.1186/s12931-020-01593-9 (PMC7734742; doi:10.1186/s12931-020-01593-9)
Supplement: Supplementary file 1 — Additional file 1: Table S1. Antibodies conjugated fluorophores, clone and supplier. Figure S1. FACS gating strategy. Table S2. Flow cytometry analysis of activated and regulatory T cells in BAL fluid, given in percent. Table S3. Flow cytometry analysis of activated and regulatory T cells in BAL fluid, given in cells/ml × 102. Table S4. Differential cell counts of leukocytes of in BAL fluid, given in number of cells/ml × 104. [file 12931_2020_1593_MOESM1_ESM.pdf]

Additional file 1

Table 1: Antibodies conjugated fluorophores, clone and supplier

Antibodies conjugated fluorophores	Clone	Supplier
CD45 Fluorescein isothiocyanate / CD14 Phycoerytrin (Simultest)	CD45: 2D1 / CD14: MΦP9	Becton Dickinson (San Jose, CA, USA)
CD3 Allophycocyanin	SK7	Becton Dickinson (San Jose, CA, USA)
CD4 Fluorescein isothiocyanate	SK3	Becton Dickinson (San Jose, CA, USA)
CD25 Phycoerytrin-Cy5	M-A 251	Becton Dickinson (San Jose, CA, USA)
FoxP3 Phycoerytrin	236A/E7	eBioscience, Inc. (Thermo Fisher Scientific, Sweden)

Figure 1: FACS gating strategy

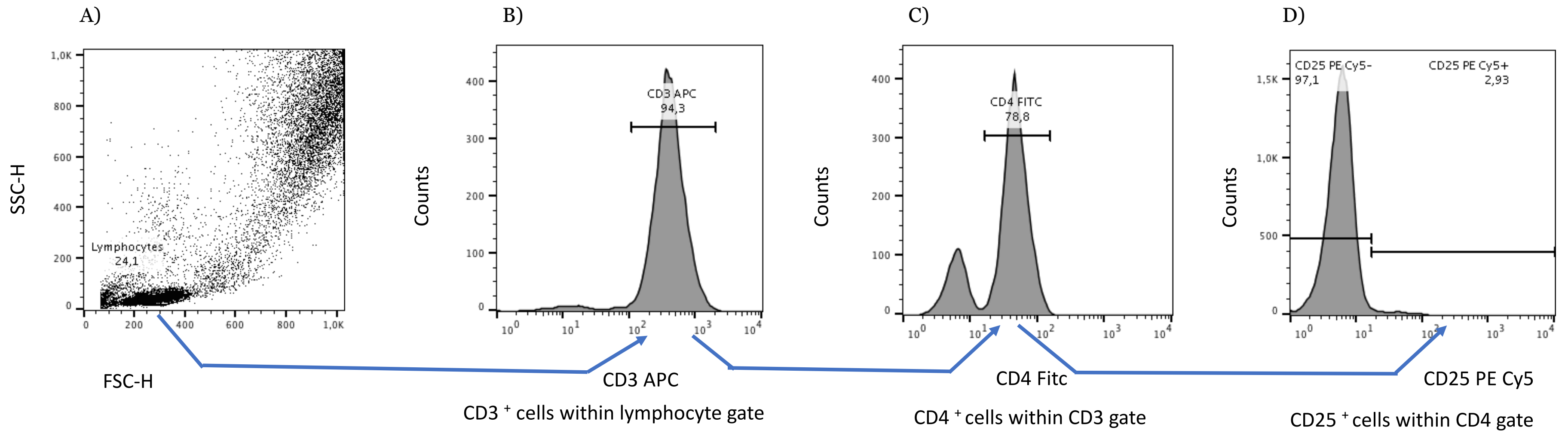

1.1 *General gating strategy*. Populations were defined in a stepwise manner using the logical operators found in the CellQuest software. In the example above, panel B shows CD3⁺ events within the lymphocyte gate. Panel C shows CD4⁺ events within the CD3 gate (logical operators: CD3⁺ *AND* lymphocyte gate); and panel D shows CD25⁺ events within the CD4 gate (logical operators: CD4⁺ *AND* CD3⁺ *AND* lymphocyte gate).

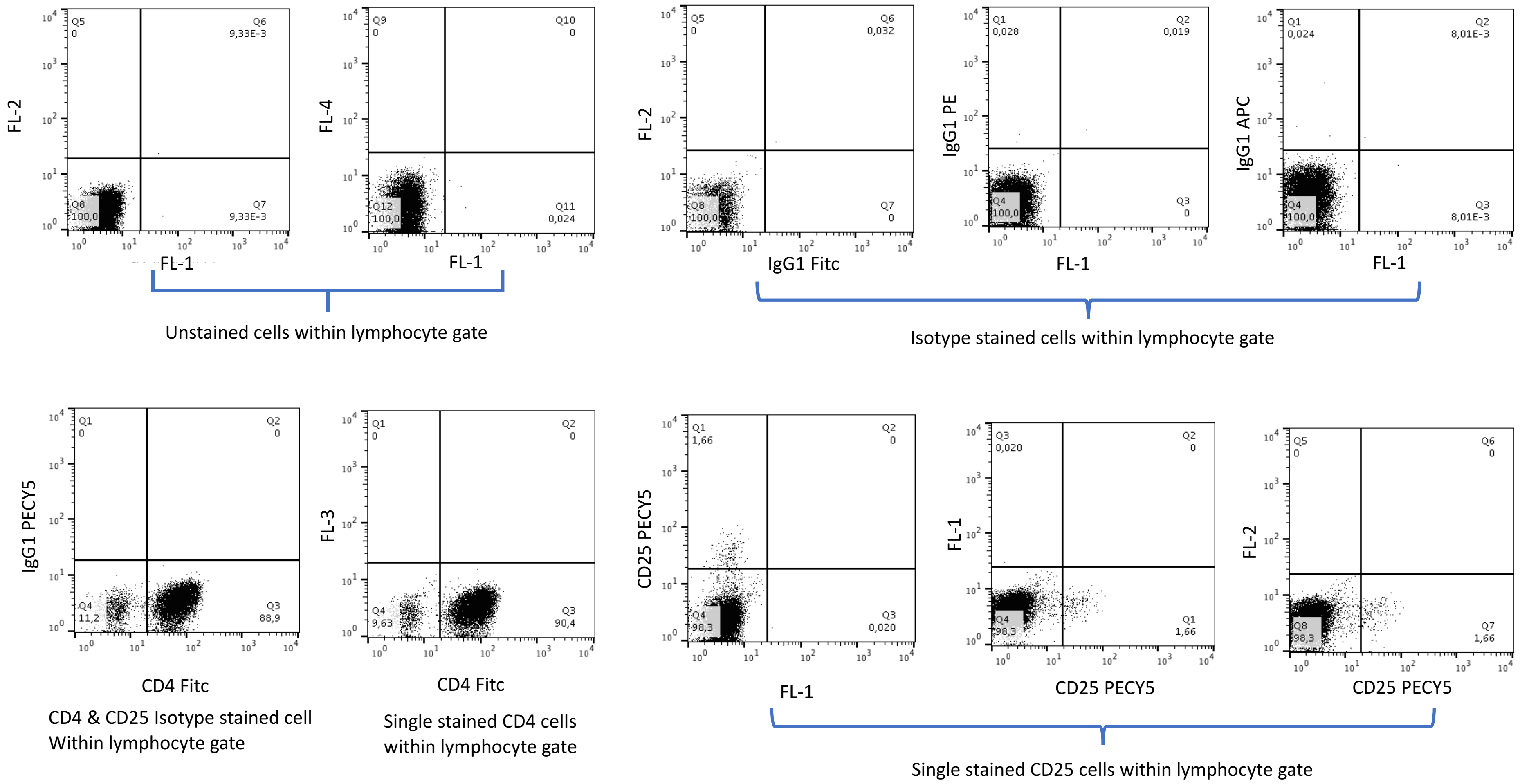

1.2 *Example plots of controls used to confirm quadrant placement and specificity of antibody expression within respective cell population*. As shown above, unstained cells, isotype stained cells, single staining of parent cells and single stained cells – all within the lymphocyte gate – were used when setting the quadrants for (in this example) the $CD25^{+}$ population.

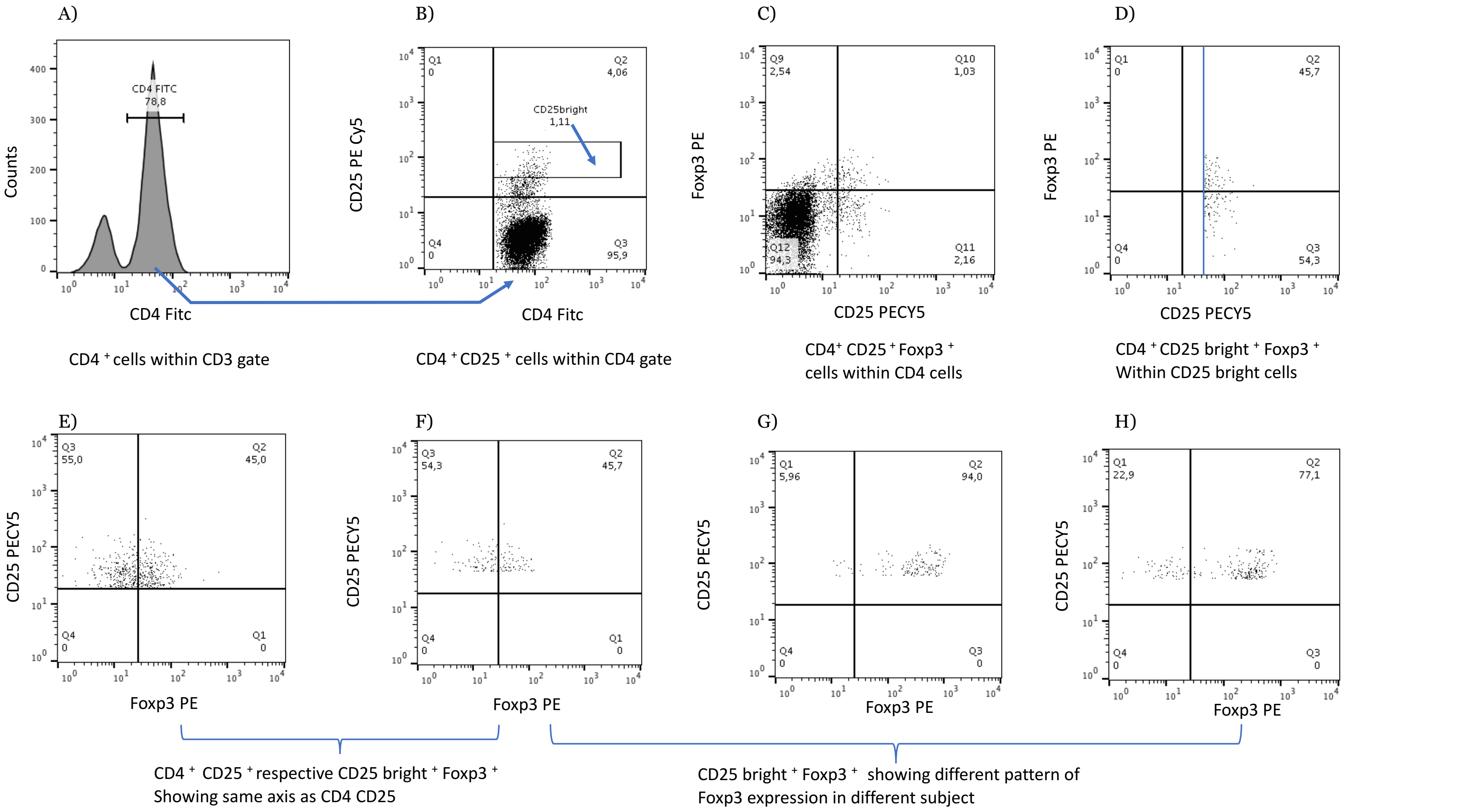

1.3 *Example plots for the FoxP3⁺ population.* A) $CD4^+$ cells within the CD3 gate. B) $CD4^+$ $CD25^+$ cells within the CD4 gate. Box indicating the $CD25^{bright}$ population. Panels C and D) Comparison plots, CD25 vs FoxP3: $CD4^+$ $CD25^+$ $FoxP3^+$ within CD4 cells; and $CD4^+$ $CD25^{bright}$ $FoxP3^+$ within $CD25^{bright}$ cells respectively. Panels E and F) Comparison of $CD4^+$ $CD25^+$ and $CD4^+$ $CD25^{bright}$ $FoxP3^+$, same axis as panel B. Panels F, G and H) Different pattern of FoxP3 expression in relation to $CD25^{bright}$ in different subjects.

Table 2: Flow cytometry analysis of activated and regulatory T cells in BAL fluid, given in percent

**Part 1: Characterizing the inflammation**				
	**COPD**<br><br>**n = 18**	**Ever-smokers with normal LF**<br>**n = 13**	**Non-smokers with normal LF**<br>**n = 15**	**p**
Activated T helper cells	2.0 (1.4-3.6)	1.4 (1.2-3.0)	1.4 (1.0-2.4)	NS
$FoxP3^+$ regulatory T cells	73 (60-82)	78 (55-86)	73 (61-79)	NS

Data are given as median with IQR. Percentage calculated out of gated cells, see main article Table 2. Statistical comparisons between the three groups were made using Kruskal Wallis test and a p-value < 0.05 was considered significant. *NS*: Not significant.

**Part 2: Separating the effect of smoking from that of COPD**				
	**COPD**<br>**current smokers**<br>**n = 10**	**COPD**<br>**ex-smokers**<br>**n = 8**	**Ex-smokers with normal LF**<br>**n = 11**	**p**
Activated T helper cells	2.1 (1.6-4.6)	1.4 (1.05-3.0)	1.3 (1.1-1.5)	NS
$FoxP3^+$ regulatory T cells	75 (63-81)	70 (48-90)	78 (63-87)	NS

Data are given as median with IQR. Percentage calculated out of gated cells, see main article Table 2. Statistical comparisons between the three groups were made using Kruskal Wallis test and a p-value < 0.05 was considered significant. *NS*: Not significant.

**Part 3: COPD and a rapid/non-rapid decline in lung function**			
	**COPD**<br>**rapid decline in lung function**<br>**n = 11**	**COPD**<br>**non-rapid decline in lung function**<br>**n = 7**	**p**
Activated T helper cells	1.9 (1.2-2.2)	2.9 (1.4-4.8)	NS
$FoxP3^+$ regulatory T cells	64 (58-80)	86 (67-91)	**p = 0.019**

Data are given as median with IQR. Statistical comparisons between the two groups were made using the Mann-Whitney U-test and a p-value < 0.05 was considered significant. *NS*: Not significant.

Table 3: Flow cytometry analysis of activated and regulatory T cells in BAL fluid, given in cells/ml x 10^2

**Part 1: Characterizing the inflammation**				
	**COPD**<br><br>**n = 18**	**Ever-smokers with normal LF**<br>**n = 13**	**Non-smokers with normal LF**<br>**n = 15**	**p**
Activated T helper cells	1.6 (0.81-2.7)	1.3 (1.0-2.9)	2.2 (1.5-3.5)	NS
FoxP3⁺ regulatory T cells	1.1 (0.49-2.2)	1.1 (0.66-2.2)	1.5 (0.84-2.2)	NS

Data are given as median with IQR. Percentage calculated out of gated cells, see main article Table 2. Statistical comparisons between the three groups were made using Kruskal Wallis test and a p-value < 0.05 was considered significant. *NS*: Not significant.

**Part 2: Separating the effect of smoking from that of COPD**				
	**COPD current smokers**<br>**n = 10**	**COPD ex-smokers**<br>**n = 8**	**Ex-smokers with normal LF**<br>**n = 11**	**p**
Activated T helper cells	1.6 (0.79-3.8)	1.6 (0.80-2.4)	1.3 (0.95-2.7)	NS
FoxP3⁺ regulatory T cells	1.1 (0.47-3.1)	1.0 (0.50-1.9)	0.90 (0.55-1.7)	NS

Data are given as median with IQR. Percentage calculated out of gated cells, see main article Table 2. Statistical comparisons between the three groups were made using Kruskal Wallis test and a p-value < 0.05 was considered significant. *NS*: Not significant.

**Part 3: COPD and a rapid/non-rapid decline in lung function**			
	**COPD rapid decline in lung function**<br>**n = 11**	**COPD non-rapid decline in lung function**<br>**n = 7**	**p**
Activated T helper cells	1.6 (1.1-2.4)	1.7 (0.58-4.2)	NS
FoxP3⁺ regulatory T cells	1.1 (0.66-1.4)	1.5 (0.39-3.6)	NS

Data are given as median with IQR. Statistical comparisons between the two groups were made using the Mann-Whitney U-test and a p-value < 0.05 was considered significant. *NS*: Not significant.

Table 4 Differential cell counts of leukocytes of in BAL fluid, given in number of cells/ml x10^4

Part 1: Characterizing the inflammation				
	**COPD**<br>**n = 19**	**Ever-smokers with normal LF**<br>**n = 15**	**Non-smokers with normal LF**<br>**n = 15**	**p**
Macrophages	17 (11-27)	14 (9.3-31)	11 (8.6-16)	NS
Neutrophils	0.18 (0.088-0.81)	0.11 (0.049-0.23)	0.1 (0.044-0.17)	NS
Lymphocytes	1.8(0.78-2.6)	1.6(1.3-3.6)	2.1(1.4-3.8)	NS
Eosinophils	0.077 (0-0.37)	0.022 (0-0.2)	0.027 (0-0.044)	NS
Mast cells	0.029 (0-0.11)	0.0043 (0-0.049)	0.017 (0.0056-0.02)	NS

Data are given as median with IQR. Statistical comparisons between the three groups were made using Kruskal Wallis test and a p-value < 0.05 was considered significant. *NS*: Not significant.

Part 2: Separating the effect of smoking from that of COPD				
	**COPD current smokers (CCuS)**<br>**n = 10**	**COPD ex-smokers (CExS)**<br>**n = 9**	**Ex-smokers with normal LF (ExS)**<br>**n = 12**	**p**
Macrophages	22 (19-34)	11 (8.4-15)	13 (8.7-17)	**p = 0.003**<br>CCuS vs CExS
Neutrophils	0.17 (0.081-0.72)	0.18 (0.065-1.5)	0.11 (0.053-0.22)	NS
Lymphocytes	1.8 (0.92-2.7)	1.8 (0.75-2.6)	1.9 (0.83-3.4)	NS
Eosinophils	0.11 (0-0.24)	0.068 (0.013-0.44)	0.02 (0-0.032)	NS
Mast cells	0.093 (0.022-0.13)	0.0051 (0-0.029)	0.0014 (0-0.059)	NS

Data are given as median with IQR. Statistical comparisons between the three groups were made using Kruskal Wallis test and a p-value < 0.05 was considered significant. If the Kruskal Wallis test indicated significance, the Mann-Whitney U-test was used for post hoc analysis for comparison of CExS vs CCuS and CExS vs ExS. A p-value < 0.05 was considered significant. *NS*: Not significant.

Part 3: COPD and a rapid/non-rapid decline in lung function			
	**COPD rapid decline in lung function**<br>**n = 11**	**COPD non-rapid decline in lung function**<br>**n = 8**	**p**
Macrophages	21 (13-27)	13 (8.3-26)	NS
Neutrophils	0.18 (0.094-0.81)	0.13 (0.038-0.88)	NS
Lymphocytes	1.9 (1-3.2)	1.6 (0.71-2.1)	NS
Eosinophils	0.068 (0-0.13)	0.12 (0.0063-0.52)	NS
Mast cells	0.065 (0.022-0.11)	0.0049 (0-0.095)	NS

Data are given as median with IQR. Statistical comparisons between the two groups were made using the Mann-Whitney U-test and a p-value < 0.05 was considered significant. *NS*: Not significant.
